# Analysing potent biomarkers along phytochemicals for breast cancer therapy: an in silico approach

**DOI:** 10.1007/s10549-023-07107-7

**Published:** 2023-09-20

**Authors:** Nivruthi Shekar, Paton Vuong, Parwinder Kaur

**Affiliations:** UWA School of Agriculture and Environment, University of Western Australia, 35-Stirling Highway, Perth, WA 6009 Australia

**Keywords:** Cancer cells, Herbal compounds, Phytochemicals, Docking, In silico analysis

## Abstract

**Purpose:**

This research focused on the identification of herbal compounds as potential anti-cancer drugs, especially for breast cancer, that involved the recognition of Notch downstream targets NOTCH proteins (1–4) specifically expressed in breast tumours as biomarkers for prognosis, along with P53 tumour antigens, that were used as comparisons to check the sensitivity of the herbal bio-compounds.

**Methods:**

After investigating phytochemical candidates, we employed an approach for computer-aided drug design and analysis to find strong breast cancer inhibitors. The present study utilized *in silico* analyses and protein docking techniques to characterize and rank selected bio-compounds for their efficiency in oncogenic inhibition for use in precise carcinomic cell growth control.

**Results:**

Several of the identified phytocompounds found in herbs followed Lipinski’s Rule of Five and could be further investigated as potential medicinal molecules. Based on the Vina score obtained after the docking process, the active compound Epigallocatechin gallate in green tea with NOTCH (1–4) and P53 proteins showed promising results for future drug repurposing. The stiffness and binding stability of green tea pharmacological complexes were further elucidated by the molecular dynamic simulations carried out for the highest scoring phytochemical ligand complex.

**Conclusion:**

The target-ligand complex of green tea active compound Epigallocatechin gallate with NOTCH (1–4) had the potential to become potent anti-breast cancer therapeutic candidates following further research involving wet-lab experiments**.**

**Supplementary Information:**

The online version contains supplementary material available at 10.1007/s10549-023-07107-7.

## Introduction

Breast cancer as the most prevalent malignant type of cancer is the main driver of cancer-related deaths globally [1]. According to statistics, 2.3 million were diagnosed with breast cancer in 2020 globally, and the incidence rate of breast cancer between 2014 and 2018 has been gradually increasing by 0.5% annually each year [2, 3]. Surgical intervention is the primary form of treatment for breast cancer, whilst other treatments include chemotherapy, radiation, endocrine therapy, targeted therapy, and immunotherapy [4–6]. Notable alternatives are secondary metabolites from plants, which present accessible and effective natural substances with a range of bioactive constituents, including anti-cancer effects [7]. Numerous anti-cancer medications were discovered by years of intensive research into the underlying molecular mechanism of malignancies. Regrettably, most medications have substantial side effects, such as toxicity and therapeutic resistance [8–10]. By 2040, it is anticipated that there would be 16.4 million cancer-related deaths and 29.5 million new instances of cancer annually [11, 12]. Chemotherapy and radiotherapy are two examples of conventional cancer therapies with negative procedure consequences, such as functional impacts causing fatigue, nausea, anaemia, loss of oxygen, and emotional impact, such as stress [13–15].

Finding novel anti-cancer medications that are less toxic and have better therapeutic effects are now crucial. There has been a huge shift away from chemically derived pharmaceuticals and towards substances based on natural products, and many scientific works have begun to investigate the anti-cancer potential of phytochemicals [16]. By altering signalling pathways, the phytochemicals obtained from a variety of medicinal plants can stop tumour growth, aggressiveness, and motility of cancerous cells [17–23]. As a result, phytochemicals have received a lot of interest in recent years as potential agents for cancer therapy [24].

The basic Notch signalling system is essential for cell proliferation, division, and cell fate determination and is evolutionarily conserved across many organisms [25, 26]. Important processes like breast growth, healthy hematopoiesis, and colorectal epithelial maturation are affected by Notch [26–30]. Malignant growth is linked to the dysregulation of the Notch signalling cascade, with other epithelial malignancies and chronic intestinal inflammation also linked to the Notch signalling pathway [30–33]. Depending on the type of tumour, Notch signalling pathways have been observed to either enhance or hinder tumour growth through angiogenesis, cell cycle progression, differentiation, cellular metabolism, and immunological function [34–37]. According to a recent review, Notch receptors are intimately involved in the Notch signalling pathways oncogenic role in haematological malignancies, lung adenocarcinoma, breast cancer, and ovarian cancer [38]. Despite numerous publications linking Notch signalling to breast cancer, no clinical research on patients with breast cancer has been performed, with limited studies on the relationship between Notch-related gene expression and the clinical characteristics of breast cancer patients [39]. The improper functioning of the Notch signalling pathway has been associated with a range of ailments, such as cancer, cardiovascular disorders, and neurological conditions [40–42]. These diseases can be attributed to the disruption of normal cellular processes, resulting in abnormal growth, development, and function of various bodily systems [43]. As such, understanding the intricacies of Notch-based signalling is critical for the development of effective treatments and prevention strategies for these debilitating conditions. The Notch receptor consists of several domains, including the extracellular domain, transmembrane domain, and intracellular domain. The Notch receptor proteins (Notch 1, Notch 2, Notch 3, and Notch 4) all share a similar structure, consisting of various domains that contribute to their function [44, 45]. One of the key domains involved in ligand binding and activation is the extracellular domain, which contains multiple epidermal growth factor (EGF)-like repeats [46]. These repeats are responsible for interacting with the ligands of the Notch pathway and triggering downstream signalling events [47]. Phytochemicals can potentially interact with these EGF-like repeats, influencing the activation of the Notch receptor [48]. In cancer treatment, dysregulation of Notch signalling promotes survival and proliferation of cancerous cells, making inhibition of specific domains like the extracellular domain that binds to ligands beneficial for therapeutic purposes [49]. The extracellular domain is a critical component for ligand recognition and receptor activation, leading to the release of Notch intracellular domain (NICD) and downstream cellular responses. Notch 1–4 receptors, expressed in various tissues, play distinct roles in development, homeostasis, and disease [50] with significant potential for therapeutic applications across various health conditions by inhibiting specific domains of the Notch receptor. However, research in this area is still work in progress, and any potential therapeutic interventions still require thorough testing and validation [51].

The Notch signalling pathway has gained significant attention in breast cancer therapy due to its multifaceted role in tumour development, progression, and treatment resistance [52, 53]. The four Notch receptors exhibit distinct functions in breast cancer, making them potential therapeutic targets. Notch 1 plays a crucial role in breast cancer by promoting cell proliferation and survival [54]. It is commonly overexpressed in various breast cancer subtypes, leading to chemotherapy resistance and stem cell preservation [55]. Targeting Notch 1 can hinder tumour growth, enhance chemotherapy efficacy, and reduce stem cell-like properties [56]. Notch 2’s role in breast cancer is not as well understood as Notch 1, but has been known to impact cell fate decisions and differentiation in the mammary gland [57]. Notch 2 may regulate estrogen receptor signalling in hormone receptor-positive breast cancer and modulating its activity could impact hormone responsiveness and contribute to targeted therapies [58]. Notch 3 is often upregulated in HER2-positive and triple-negative breast cancers and is associated with aggressive phenotypes and poor outcomes [59]. Notch 3 signalling can enhance cell survival, invasion, and metastasis of cancerous cells. Suppressing Notch 3 has been investigated to limit metastasis and enhance cell response to chemotherapy [60, 61], as well as affect the tumour microenvironment and decrease activation of cancer-associated fibroblasts [62]. Notch 4’s role in breast cancer is not well-defined compared to other Notch receptors, but has been linked to angiogenesis and vascularization, which may influence tumour growth and metastasis [63]. Expression of Notch 4 is associated with the basal-like subtype of breast cancer. Its involvement in promoting endothelial cell survival suggests a possible role in therapies that inhibit angiogenesis [64]. Notch 1–4 receptors have intricate and context-dependent roles in the development and progression of breast cancer. Targeting these receptors and the Notch signalling pathway shows promise in creating innovative therapeutic approaches in breast cancer therapy [51]. However, further research is necessary to understand the complex interactions and consequences of modulating Notch signalling in breast cancer cells.

Molecular categories for oncology biomarkers include different types, such as genetic, epigenetic, proteins, glycoproteins, receptors, and hormones [28, 31–33, 40]. The classification of tumour biomarkers includes pharmacodynamic, prognostic, diagnostic, and predictive types. Since their sensitivity and specificity offer excellent accuracy, protein biomarkers have been heavily relied upon in early diagnosis and treatment [65, 66]. Other classifications based on clinical characteristics are Type 0, Type I, and Type II biomarkers. Type 0 biomarkers linked to well-known clinical signs are used to track the diseases' natural histories. On the other hand, type I biomarkers are associated with the efficiency of pharmacological treatments. Secondary biomarkers, or type II biomarkers, were developed to take the place of clinical goals [67]. The estrogen receptor (ER), progesterone receptor (PR), and human epidermal growth factor receptor 2 (HER2) are well-known biomarkers for predicting the subtype, prognosis, and treatment options for breast cancer patients [68–71, 78]. Recurrence rates and treatment effectiveness are assessed using biomarkers, such as CA15-3, HER2, CA 27.29, estrogen/progesterone receptors, urokinase plasminogen activator, and plasminogen activator inhibitor (PAI-1) [71, 79, 80].

The tumour protein Notch (1–4) have had limited research in breast cancer-related studies for drug discovery. Notch receptors when downregulated appear to have suppressive activity towards breast cancer [72–76]. As such, Notch receptor mutations are linked to poor prognoses and contribute to the pathophysiology of solid tumours and human haematological malignancies [52, 53]. A popular option for anti-cancer treatments is P53, which is mutated in most breast cancer cases [77]. Mutated P53 is another potential biomarker and therapeutic target for people with breast cancer, with a number of pharmaceuticals on the market that can restore the wild-type features of mutant P53 proteins with demonstrated anti-cancer efficacy [54, 55]. Whilst P53 was once thought to be “non-druggable,” drugs that specifically target mutated P53 have recently appeared on the market [56, 57, 81]. A high level of complexity is involved in understanding the pharmacokinetics of protein biomarkers, with the development of suitable therapeutic treatments requiring substantial time and resources invested into the clinical decision-making [58, 59, 82, 83].

The initial stages of this study investigated various herbal constituents and their potential applications in the prevention of chronic ailments, with a focus on anti-cancer activity. Recent articles on the topic of herbs and spices that potentially exhibited anti-cancer and anti-tumorigenic properties were surveyed and studied. After careful collation of literature, a proposed investigation of selected phytochemicals was performed, with a specific focus on their potential to prevent the onset of breast cancer. Research has shown that Epigallocatechin gallate, an active compound present in Green Tea amongst other phytocompounds can induce apoptosis and inhibit the growth of several cancer types, such as colon, kidney, breast, brain cancers, and leukaemia both in vivo and in vitro [84]. It has also shown that Epigallocatechin gallate has the ability to restrict the expansion of cancer cells, particularly breast cancer cells [85, 86]. Furthermore, a study on Asian-American women revealed that frequent consumption of green tea was linked to a lower risk of developing breast cancer [87]. Piperine from black pepper shown to inhibit the growth and motility of triple-negative breast cancer cells whilst leaving normal breast cells unaffected, but the clinical use of Piperine is limited by its hydrophobic nature and poor aqueous solubility [88, 89]. Ginger and its main bioactive compound, gingerol, have shown cytotoxic effects on various cancer types, including breast, cervical, colorectal, leukaemia, liver, lung, nasopharyngeal, ovarian, prostate, and retinoblastoma [90–92]. Black cumin’s derivative Thymoquinone has been found to have positive effects on reducing carcinogenic effects in rats with mammary carcinoma when combined with melatonin and retinoic acid. Derivatives of black cumin have also been tested and shown to induce cell death by apoptosis in MCF-7/Topo breast carcinoma [93].

A comprehensive analysis performed on the inhibitory effects of phytochemicals on various stages of the signalling pathway indicated how these compounds impede the Notch pathway. Epigallocatechin gallate (Green tea) is a polyphenolic compound that can interfere with the Notch signalling pathway at multiple levels [94]. It is suggested that Epigallocatechin gallate may inhibit the ligand–receptor interaction by disrupting the binding of Notch ligands (such as Jagged or Delta-like) to the Notch receptor extracellular domain. Additionally, it might influence the γ-secretase complex, which is responsible for the second cleavage of the Notch receptor, thereby preventing the release of the NICD [95]. Moreover, it could affect the translocation of NICD to the nucleus of cancer cells and its interaction with transcriptional co-activators [96]. Piperine’s inhibitory effects on the Notch pathway may involve interfering with the proteolytic cleavage of the Notch receptor. It could target the γ-secretase complex and inhibit the second cleavage step, preventing the release of NICD [88]. This action would subsequently hinder the formation of the Notch intracellular domain and its downstream transcriptional activities [97]. Gingerol’s inhibitory mechanisms on the Notch pathway may encompass modulating the ligand–receptor interaction, preventing the ligand from binding to the Notch receptor [98]. Moreover, gingerol might disrupt the γ-secretase-mediated cleavage of Notch, impairing the generation of NICD. By doing so, gingerol could attenuate the downstream signalling events driven by NICD [99]. Thymoquinone’s potential inhibition of the Notch signalling pathway might involve interfering with the ligand–receptor interaction, possibly by affecting ligand presentation or receptor binding [100]. Additionally, thymoquinone could impact the γ-secretase-mediated cleavage of Notch, thus reducing the production of NICD [101]. This action would subsequently hinder the activation of downstream Notch target proteins.

**Fig. 1:**
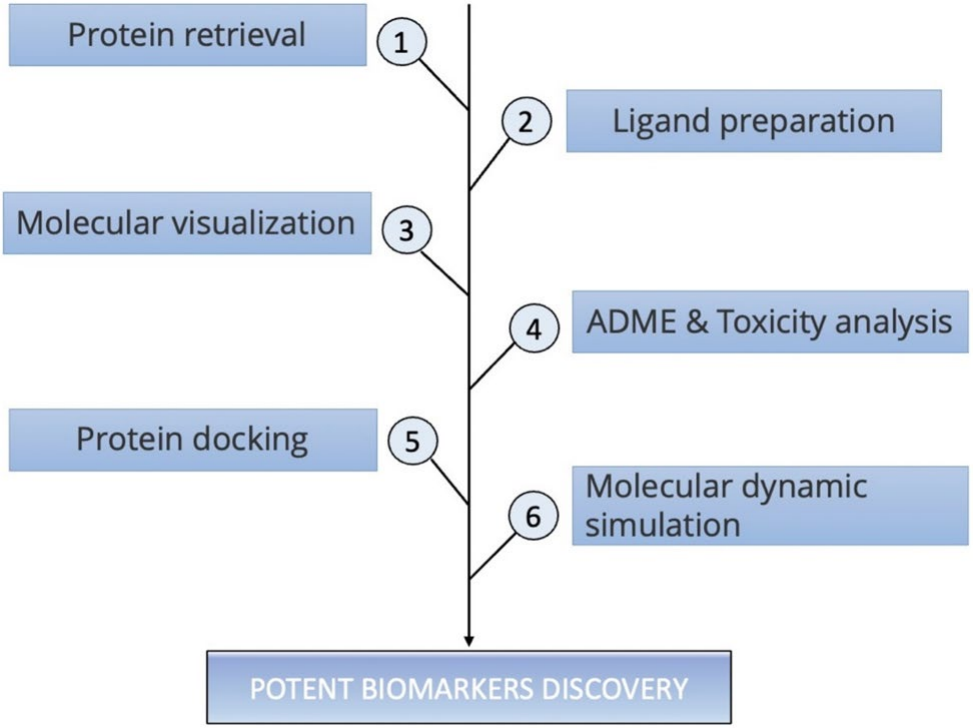
Graphical representation of the methods employed in this study

Table 1 presents a candidate list of herbs with compounds containing unique anti-cancer or anti-tumorigenic properties. Amongst these herbs, no research to date has been done to identify the anti-cancer compounds found in Green tea, Black pepper, Black cumin, and Ginger against Notch inhibitors. Due to the limited literature available, the protein–ligand complexes that are investigated in this study have been involved in fewer research avenues. We used the CB-Dock2 server for docking and ranking the target-ligand complex based on their scoring function [102]. This study performed virtual screenings of various phytochemicals with the Notch protein to identify anti-tumour compounds from plants. ADMET was then used to perform drug-likeness predictions and molecular dynamic simulation analyses. Based on docking scores, the screened compounds were subjected to molecular dynamic simulation (MDS) to understand the mechanisms of action better. Through this process, we uncovered novel phytochemicals that can be potentially employed as Notch inhibitors. Natural phytochemicals derived from plants have been found to be an effective, low-risk treatment for stage 1 breast cancer. Our research presented an in silico workflow for identifying phytochemical compounds as potential sources of anti-cancer medication candidates (Fig. 1).

**Table 1:** Phytochemical details of each herb and its anti-cancer activity

S. no	Phytochemical	Plant Family	Phytochemical family	Anti-cancer activity	Refs
1	Echinacea	Asteraceae	Flavonoids	Increases the natural killer cells	[103]
2	Garlic	Allium sativum	Sulfurones-Alliin	Interferes with tumour cell metabolism, suppressor T- cells increased	[104]
3	Turmeric	Curcuma longa	Phenols	Has inhibitory action in all phases of tumour growth	[105]
4	Burdock	Arctium lappa	Phenols	Stimulates macrophage activity, limits tumour division, preserve immune-modulatory properties	[106]
5	Green tea	Camellia sinensis	Polyphenols Flavonoids	Restricts cancer cell multiplication, induce apoptosis & necrosis	[107]
6	Ginseng	Panax ginseng	Ginsenosides	Inhibits the growth and metastasis of malignant cells, increases interferon levels. The natural killer cell cycle is enhanced, promotes the production of antibodies	[108]
7	Black cohosh	Cimicifuga racemosa	Triterpene glycosides	When combined with chemotherapy drug, has significant impacts on breast tumours	[109]
8	Flax seed	Linum usitatissimum	Lignans	Decrease of tumour growth	[110]
9	Ginger	Zingiber officinale	polyphenols	Have antiproliferative effects, proapoptotic	[111]
10	Black pepper	Piper nigrum	Piperine	High antioxidant properties, interference with signalling pathways	[112]
11	Black cumin	Nigella sativa	Flavonoid-Thymoquinone	Used as combinational agent, anti-tumour activity	[113]

## Materials and methods

### Target protein retrieval

**Fig. 2:**
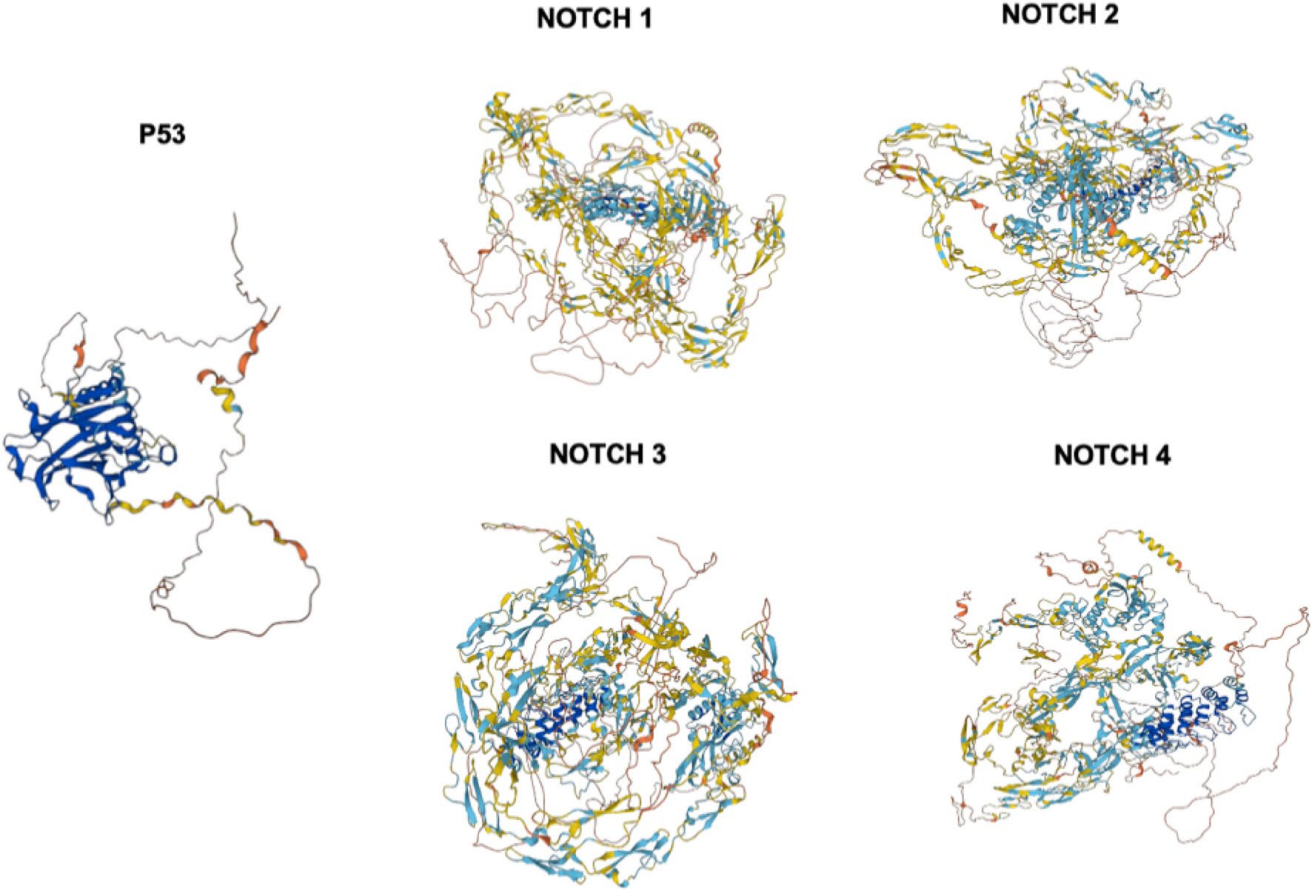
Types of proteins involved in Target retrieval. Source: Alpha fold Protein structural database

Protein homology models of P53 and NOTCH (1–4) protein sequences (Fig. 2) were retrieved from the Alpha fold homology modelling server (https://alphafold.ebi.ac.uk/). Tumour suppressor protein TP-53 or (P53), neurogenic locus notch homolog protein 1 (NOTCH1), neurogenic locus notch homolog protein 2 (NOTCH2), neurogenic locus notch homolog protein 3 (NOTCH3), and neurogenic locus notch homolog protein 4 (NOTCH4) were retrieved using the Uniprot ID P53-P04637, NOTCH1-P46531 NOTCH2-QO4721, NOTCH3-Q9UM47, and NOTCH4-Q99466 [114]. Amino acid (aa) sequences for proteins 393-P53, 2551-NOTCH1, 2471-NOTCH2, 2321-NOTCH3, and 2001-NOTCH4 derived from Homo sapiens (humans) were recovered.

### Preparation of ligands

**Fig. 3:**
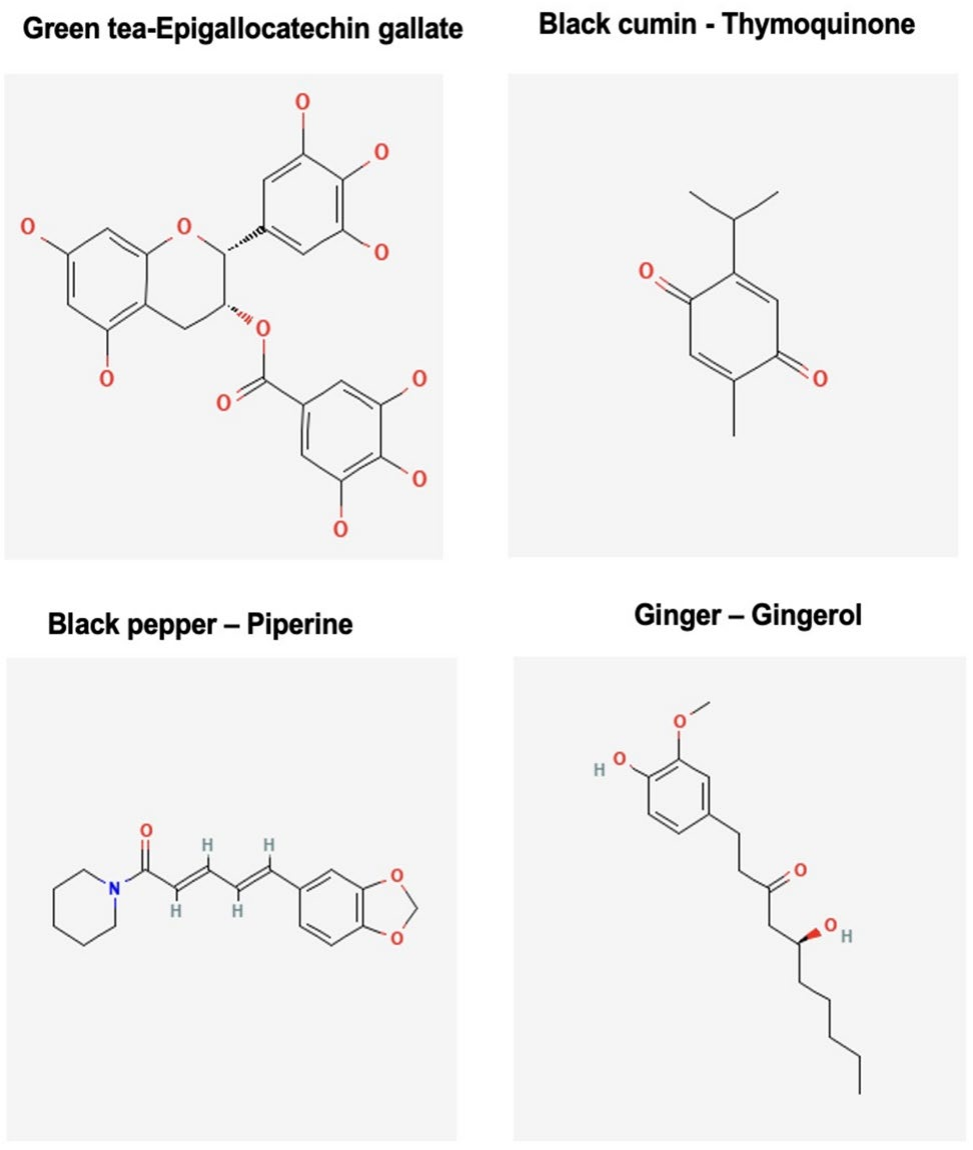
Types of herbs and their active bio-compounds 3D conformers retrieved from PubChem database. Source: PubChem database

Research papers based on Ayurveda classical books were scrutinized to compile a comprehensive list of medicinal plants known for vitality and immune enhancing characteristics, which may reflect potential anti-cancerous effects, to guide the selection of herbal bio-compounds [115–120]. Following these stipulations, phytochemicals were searched via the IMPPAT, PubChem, and ChEMBL databases, with four herbal phytochemicals shortlisted after a thorough literature survey using google scholar and PubMed databases (Fig. 3) [121, 122]. These ligands were then subject to further molecular docking and other downstream analyses. Phytochemical candidates were collected from the PubChem database (https://pubchem.ncbi.nlm.nih.gov/) in 3D SDF (Structural data file) format [20, 78, 79]. The files for the protein targets P53 and Notch 1–4 were acquired from the Alpha fold protein structural database. 3D conformers of phytochemicals (Epigallocatechin gallate CID-65064, Piperine CID-638024, Gingerol CID-442793, and Thymoquinone CID-10281), henceforth referred to as ligands, were downloaded from PubChem database (https://pubchem.ncbi.nlm.nih.gov/) (accessed on 14th March 2023) in .SDF format.

### Toxicity assessment

The ADMET server (http://lmmd.ecust.edu.cn/admetsar2) was used to determine the pharmacokinetic parameters as well as the efficacy of various synthetic and phytochemical-based medications. To assist in predicting the ADMET parameters, we also utilized the admetSAR server (http://lmmd.ecust.edu.cn/admetsar2) [123, 124]. The admetSAR server also provided predictions on numerous factors, including toxicity, carcinogenicity, and LD50 values, as well as the Blood–Brain Barrier (BBB), Human Intestinal Absorption (HIA), Caco-2 cell permeability, P-GP (P-glycoprotein) substrate/inhibition, and Cytochrome P450 metabolism.

### Molecular docking study

CB-Dock2, an open-source software (GUI version of Auto Dock Vina), was used to do a virtual screening of herbal plant phytochemicals by molecular docking to the active site of the receptor [124, 125]. This was performed to help screen the optimal inhibitor for P53 and Notch targets. The ligands were matched with each protein individually throughout the docking investigation, with the resulting output files used to extract the binding affinity/Vina score table. The assessment criteria for the best inhibitors predicted were phytochemicals with a stronger affinity for the target protein. Active site prediction was made using PyMol 2.5.2 molecular graphic system software by finding the active sites/binding pockets in the chosen target proteins where the ligand is most likely to bind with stable free energy [125]. PyMol was also used to predict the polar and non-polar interaction analysis of the complexes [126]. To study protein docking, an online-based blind docking server called CB-Dock was used (http://clab.labshare.cn/cb-dock/php/index.php) [127]. Instead of docking blindly throughout a protein’s whole surface, CB-Dock was set to only dock at predetermined locations. The initial step was a search for potential binding sites or cavity detection. A few prime cavities were selected for further investigation based on cavity size, as ligand-binding sites are often larger cavities [128]. The docking centre and cavity volume were predetermined based on the cavity pocket (C1 to C5) it was assigned to. The bound poses were reranked based on the vina score after completing the docking process. The best binding site for the query ligand and the first conformation posture based on the highest negative vina score was regarded as the optimum binding posture/conformation. The amino acid residues of each binding conformation was displayed after the docking session is completed.

### Molecular dynamics simulation

Using UCSF CHIMERA 1.17, an extensible molecular modelling system, the best-docked protein target and ligand complexes were refined and put through a molecular dynamics simulation (MDS) study. Protein complexes stored as .pdb complex files were transformed into .psf files and subsequent trajectory files were obtained. These trajectory files were utilized to enhance the complex structures by minimizing solvation and neutralization. A cubic water box with transferrable intermolecular potential was used for the solvation of the complex structures, with the box’s dimensions automated. The approximate results were retrieved using generalized born molecular mechanics surface area in an explicit solvent. NVT dynamics were implemented, which maintained constants for substance quantity (*N*), volume (*V*), and temperature (*T*). The complete simulation was run in 1000 steps over 1000 ns with the Noose–Hover temperature set to 300 K. The protein-lipid parameter for proteins and the General Force Field parameter for small-molecule ligands were used as per the study conducted by Santos et al. [129] and they were used as a sample to assign topology and force field parameters [130].

## Results

### ADME and toxicity analysis

The application domain of ADME and Toxicity verification to check the bio-compounds drug likeliness was determined by six physicochemical or topological properties: molecular weight, analogP, number of atoms, number of rings, H-bond acceptors, and H-bond donors [131]. According to Lipinski’s Rule of Five, a compound should have threshold values for all six physiochemical properties [132]. The four shortlisted herbal substances underwent pharmacodynamic, physiochemical, absorption, distribution, metabolism, and excretion (ADMET) tests, as indicated in Table 2. Herbal compounds with higher gastrointestinal absorption, solubility, blood–brain barrier permeability, good logP values (partition coefficient), and those that did not break any of Lipinski’s Rule of Five (pharmaceutical properties) were listed. Of those that passed the prior parameters, herbal compounds with good bioavailability, lead-likeness scores, and superior synthetic accessibility were then chosen.

**Table 2:** Physiochemical properties of herbal compounds provided by admetSAR server

Active Biocompound	MW	ALogP	WS (logS)	H-Bond acceptor	H-Bond donor	Rotatable bonds	HIA	BBB	Human oral bioavailability	Plasma protein binding (100%)	Acute oral toxicity Log(1/mol/kg)	Applicability domain
Epigallocatechin gallate	458.38	2.23	− 3.314	11	8	3	0.8422	0.8000	0.7000	0.986	1.614	Warning
Piperine	285.34	3.00	− 3.398	3	0	3	0.9934	0.9250	0.5714	1.083	2.788	In-domain
Gingerol	294.39	3.23	− 3.234	4	2	10	0.9911	0.6750	0.7000	0.794	1.71	In-domain
Thymoquinone	164.20	1.67	− 2.006	2	0	1	0.9969	0.7000	0.6143	0.805	1.85	In-domain

*MW* molecular weight, *WS* water solubility, *HIA* human intestinal absorption, *BBB* blood–brain barrier

Following ADME analysis, phytochemicals were tested for toxicity to select compounds that did not bind to harmful receptors or demonstrate sensitive lethality. Table 3 summarizes the significant toxicity values of each herbal compound. We selected the compounds based on their bioavailability, lead-likeness scores, and easy-to-synthesize properties. After analysing their ADME (Absorption, Distribution, Metabolism, and Excretion) characteristics, we tested the chosen phytochemicals to ensure the compounds did not bind to harmful receptors or cause sensitive lethality.

**Table 3:** (a) Toxicity prediction of selected herbal compounds. (b) Acute toxicity category values from the admetSAR server

(A) Toxicity prediction of selected herbal compounds						
Phytochemicals	AMES Toxicity	Carcinogens	Acute oral toxicity	Nephrotoxicity	Biodegradability	BRCP-Breast cancer receptor protein
Epigallocatechin gallate	AMES nontoxic	Non-carcinogenic	IV	Negative	Biodegradable	Negative
Piperine	AMES nontoxic	Non-carcinogenic	III	Negative	Biodegradable	Negative
Gingerol	AMES nontoxic	Non-carcinogenic	III	Negative	Biodegradable	Negative
Thymoquinone	AMES nontoxic	Non-carcinogenic	II	Positive	Biodegradable	Negative

(B) Acute toxicity category values from the admetSAR server					
Acute Toxicity	Category I	Category II	Category III	Category IV	Category V
Oral (mg/kg)	≤ 5 (Very low)	> 5 to ≤ 50 (medium)	> 50 to ≤ 300 (medium)	> 300 to ≤ 2000 (very high)	2000 to 5000 mg/kg

### Interpretation of protein–ligand interaction

**Fig. 4:**
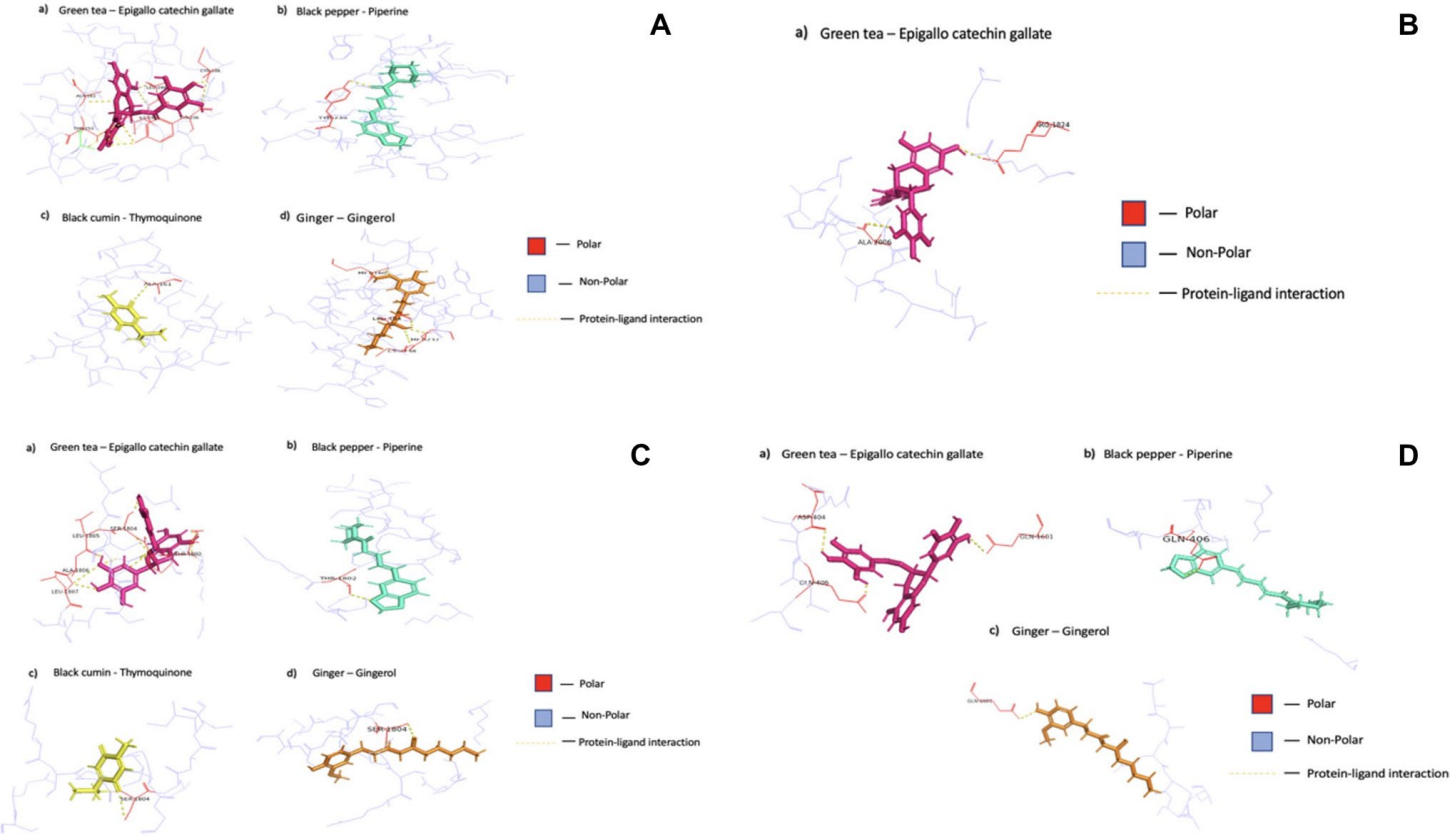
**A** Binding site residue of P53 protein with phytochemicals, **B** NOTCH1 Protein, **C** NOTCH2 protein, and **D** NOTCH4 protein

Further confirmation via interaction analysis was performed to look for contacts between the docked phytochemicals, Epigallocatechin gallate, Piperine, Gingerol, and Thymoquinone and the target proteins P53 and NOTCH (1–4) before submitting the chosen protein–ligand complexes for MDS study (Fig. 4). To determine how many contacts each phytochemical formed, we treated each of the four phytochemicals to individual protein pair docking with P53 and NOTCH (1–4). In our interaction study, we observed that complexes with strong binding affinities were those that produced the most hydrogen bonds, as shown in Fig. 4.

The unique binding characteristics of each compound was thoroughly investigated with respect to the individual receptors. To elucidate the differentiation in binding mode with the Notch 1–4 receptors, the extensive molecular docking analysis and molecular dynamics simulations study would provide us with the report of the binding modes. Our findings reveal distinct interaction patterns between each compound and the receptors, contributing to their selective binding.

1. Epigallocatechin Gallate (Green Tea)
  **Notch 1:** Epigallocatechin gallate interacts with Notch 1 through a combination of hydrogen bonding and hydrophobic interactions. Within the binding pocket of Notch 1, its hydroxyl groups engage in hydrogen bonding with specific amino acid residues, such as asparagine and serine. These interactions stabilize the complex and contribute to binding affinity. Additionally, the hydrophobic regions of Epigallocatechin gallate’s aromatic rings align with hydrophobic residues in the binding pocket, further enhancing its binding specificity to Notch 1.
  **Notch 2:** Epigallocatechin gallate binding to Notch 2 involves a distinct set of interactions. In this case, galloyl groups form hydrogen bonds with polar residues within the binding site of Notch 2, such as threonine and glutamine. The unique arrangement of phenolic rings allow for π–π stacking interactions with specific aromatic residues in Notch 2. These interactions contribute to the compound’s orientation and binding stability with this receptor.
  **Notch 3:** Binding to Notch 3 is characterized by a combination of hydrogen bonding and van der Waals interactions. Epigallocatechin gallate’s hydroxyl groups form hydrogen bonds with key residues in the binding pocket, including histidine and arginine. Furthermore, flexible molecular structure enables it to fit snugly within the binding site, forming van der Waals contacts with hydrophobic residues. This arrangement optimizes the binding affinity of Epigallocatechin gallate with Notch 3.
  **Notch 4:** Epigallocatechin gallate’s binding to Notch 4 is mediated by a mix of hydrogen bonding and electrostatic interactions. The galloyl groups engage in hydrogen bonds with specific polar residues in the binding pocket, such as tyrosine and lysine. The distribution of charges in structure complements the electrostatic environment of Notch 4’s binding site, further enhancing its binding specificity. Hydrophobic interactions between aromatic rings and hydrophobic residues contribute to the stabilization of the complex.
2. Piperine (Black Pepper)
  **Notch 1:** Piperine could potentially interact with Notch 1 through a combination of hydrogen bonding and hydrophobic interactions. Its aromatic ring structures might align with hydrophobic residues in the Notch 1 binding pocket, whilst functional groups on Piperine, such as hydroxyl or amine groups, could form hydrogen bonds with specific amino acid residues in the receptor.
  **Notch 2:** For binding to Notch 2, Piperine might form specific hydrogen bonds with polar residues within the binding site, allowing it to orient itself optimally. Additionally, its flexible molecular structure might enable it to engage in π–π stacking interactions with aromatic residues in Notch 2, contributing to its binding affinity.
  **Notch 3:** Piperine’s interaction with Notch 3 could involve a mix of hydrogen bonding and van der Waals interactions. Its functional groups might form hydrogen bonds with key polar residues in the binding pocket, whilst its non-polar regions could engage in van der Waals contacts with hydrophobic residues.
  **Notch 4:** Piperine’s binding to Notch 4 could rely on hydrogen bonding and electrostatic interactions. Its charged or polar groups might form hydrogen bonds with specific residues in the binding pocket and its distribution of charges could complement the electrostatic environment of Notch 4.
3. Gingerol (Ginger)
  **Notch 1:** Gingerol could potentially interact with Notch 1 through a combination of hydrogen bonding and hydrophobic interactions. Its aromatic ring structures might align with hydrophobic residues in the Notch 1 binding pocket, whilst functional groups on Gingerol, such as hydroxyl or methoxy groups, could form hydrogen bonds with specific amino acid residues in the receptor.
  **Notch 2:** For binding to Notch 2, Gingerol might form specific hydrogen bonds with polar residues within the binding site, allowing it to orient itself optimally. Its flexible molecular structure might enable it to fit into the binding pocket and establish interactions with residues critical for Notch 2 binding.
  **Notch 3:** Gingerol’s interaction with Notch 3 could involve a mix of hydrogen bonding and van der Waals interactions. Its functional groups might form hydrogen bonds with key polar residues in the binding pocket and its non-polar regions could engage in van der Waals contacts with hydrophobic residues.
  **Notch 4:** Gingerol’s binding to Notch 4 could rely on hydrogen bonding and electrostatic interactions. Its charged or polar groups might form hydrogen bonds with specific residues in the binding pocket and its distribution of charges could complement the electrostatic environment of Notch 4.
4. Thymoquinone (Black Cumin)
  **Notch 1:** Thymoquinone could potentially interact with Notch 1 through a combination of hydrogen bonding and hydrophobic interactions. Its aromatic ring structures might align with hydrophobic residues in the Notch 1 binding pocket, while functional groups on thymoquinone, such as hydroxyl or carbonyl groups, could form hydrogen bonds with specific amino acid residues in the receptor.
  **Notch 2:** For binding to Notch 2, Thymoquinone might form specific hydrogen bonds with polar residues within the binding site, allowing it to orient itself optimally. Its molecular structure might enable it to engage in π–π stacking interactions with aromatic residues in Notch 2, contributing to its binding affinity.
  **Notch 3:** Thymoquinone’s interaction with Notch 3 could involve a mix of hydrogen bonding and van der Waals interactions. Its functional groups might form hydrogen bonds with key polar residues in the binding pocket and its non-polar regions could engage in van der Waals contacts with hydrophobic residues.
  **Notch 4:** Thymoquinone’s binding to Notch 4 could rely on hydrogen bonding and electrostatic interactions. Its charged or polar groups might form hydrogen bonds with specific residues in the binding pocket and its distribution of charges could complement the electrostatic environment of Notch 4.

Specific binding interactions between Piperine and Notch receptors have not been extensively studied or reported in the scientific literature. However, explanations based on general molecular interactions can be given. Research on Piperine binding has been limited, thus we aimed to explore how Piperine might differentiate in binding mode with Notch receptors.

The binding interactions between the ligands and the proteins visualized via PyMol Visualizer Tool showed better interaction with the P53 Protein (Uniprot ID- P04637). P53-Green tea shows polar contact with the ligand at ALA161, LEU194, SER-215, THR253, CYS238, TYR-236, and ILE-19, whereas P53-Black pepper interaction yielded contact at TYR-236, P53-Black cumin at ALA161, and P53-Ginger interaction shows contact at MET-160, LEU194, MET-237, and CYS238. Protein–ligand polar contact with amino acids was observed with the NOTCH2 protein, but no hydrogen bonds were visible. NOTCH2-Green tea non-polar contacts were seen in amino acids THR-119 and THR-121. NOTCH2-Black pepper pair amino acids were THR-121, CYS-120, and THR-119. NOTCH2-Black cumin pair and their non-polar amino acids were THR-121 and THR-119. NOTCH2-Ginger pair showed contact signs at THR-119, CYS-120, and THR-121. NOTCH3 protein did not have any protein–ligand interaction, which was consistent with other analyses in the current literature. NOTCH4-Green tea complex showed hydrogen bonds at sites ASP404, GLN406, and GLN1601. NOTCH4-Black pepper yielded contact in GLN406, and NOTCH4-Ginger contact was seen in GLN1601. The consolidated data for the amino acid residues are presented in Table 4. Since these are known to stabilize protein complexes and are important for molecular recognition processes, Epigallocatechin gallate was notable for producing many hydrogen bonds. Due to the conformational space that Piperine, Gingerol, and Thymoquinone inhabited, they produced a few hydrogen bonds. The interactions between the target proteins P53 and NOTCH (1–4) and the shortlisted ligands are shown in Fig. 4.

**Table 4:** Consolidated results for protein–ligand interaction

Protein–Ligand Complex	Hydrogen bond residues
P53—Green tea	ALA161, LEU194, CYS238, TYR236, ILE195, THR253
P53—Black pepper	TYR236
P53—Ginger	MET-160, LEU194, CYS238, MET-237
P53—Black cumin	ALA161
Notch 1—Green tea	ALA2006, ARG 1824
Notch 2—Green tea	LEU1807, ALA1806, LEU1805, SER1804, THR1802
Notch 2—Black pepper	THR1802
Notch 2—Black cumin	SER1804
Notch 2—Ginger	SER1804
Notch 4—Green tea	ASP404, GLN406, GLN1601
Notch 4—Black pepper	GLN406
Notch 4—Ginger	GLN1601

*Notch 3 proteins had no bonds involved with the phytochemicals

It is important to note that these explanations are speculative and based on general knowledge of molecular interactions. The actual binding modes of Piperine, Gingerol, and Thymoquinone with Notch receptors would require rigorous experimental investigation, such as molecular docking studies, X-ray crystallography, or NMR spectroscopy, to provide accurate details about the binding interactions. In summary, our computational analyses have revealed that the compounds exhibit varying binding modes with the Notch 1–4 receptors due to their distinct chemical structures and interactions. We believe these findings shed light on the molecular basis of the compounds’ selectivity and efficacy towards different Notch receptors.

### Protein docking

**Fig. 5:**
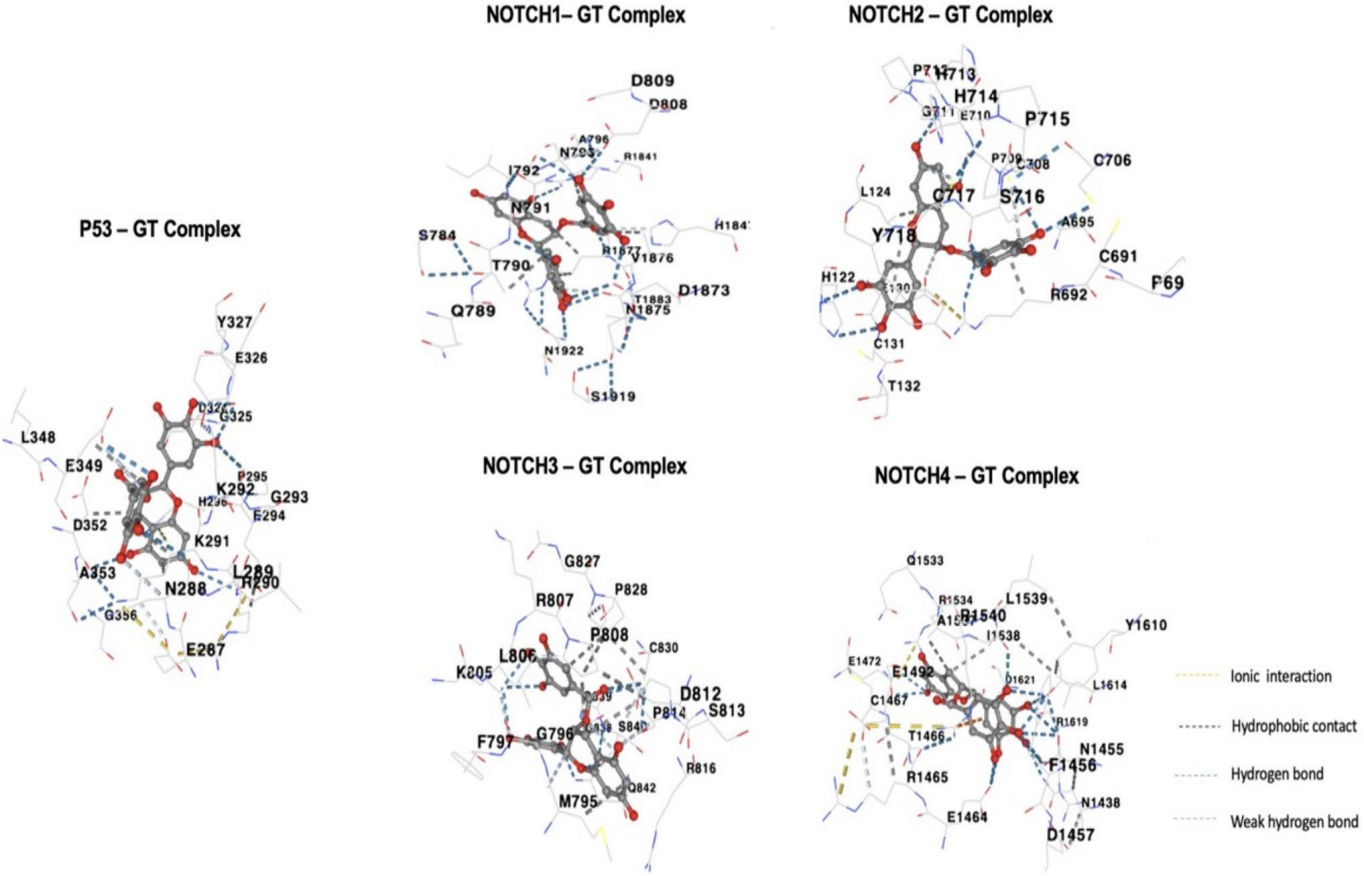
Docked poses of Green tea ligand Epigallocatechin gallate with P53 and NOTCH 1–4 proteins. Yellow-dotted lines represent Ionic/electrostatic interactions, teal-dotted lines represent strong hydrogen bonds, whilst hydrophobic interactions are represented by grey-dotted lines

Four phytochemicals examined after the docking study with higher affinity/scoring function for the target proteins NOTCH 1–4 and P53 are shown in Table 3 as Epigallocatechin gallate, Piperine, Gingerol, and Thymoquinone. The phytochemicals docked to the target proteins NOTCH 1–4 and P53 as shown in Fig. 5. Our investigation into the effects of certain compounds on the Notch receptors has yielded intriguing results that could pave the way for novel therapeutic strategies. For Epigallocatechin gallate the binding pockets of Notch 1, 2, 3, and 4 form many hydrogen bonds and hydrophobic interactions with certain residues, according to our computational investigations. Notably, aromatic rings form van der Waals interactions with hydrophobic residues, whereas its hydroxyl groups form hydrogen bonds with polar amino acids. According to this binding method, Epigallocatechin gallate may interfere with crucial cleavage events and impede ligand–receptor interactions, affecting each Notch receptor’s downstream signalling cascades (refer to Fig. 5). Piperine, for instance, has been found to adopt distinct binding poses in the binding sites of Notch 1, 2, 3, and 4 (In supplementary Figs. 1–4). This is due to its flexible structure, which allows it to establish hydrogen bonds with specific polar residues and participate in π–π stacking interactions with key aromatic amino acids. The implications of these interactions are manifold; they suggest that Piperine has the potential to not just interfere with ligand binding but also cleavage events or downstream signalling processes for each Notch receptor. Similarly, our analysis of Gingerol has revealed that it interacts with the Notch receptors through a combination of hydrogen bonds and hydrophobic contacts (In supplementary Figs. 1–4). Gingerol’s binding poses suggest it may interfere with ligand recognition, cleavage, or nuclear complex formation. The interactions that Gingerol has with specific residues in the binding pockets of each Notch receptor also suggest its ability to modulate key signalling events. Finally, Thymoquinone has been found to establish binding interactions involving hydrogen bonding, electrostatic interactions, and van der Waals forces with Notch 1, 2, 3, and 4 (In supplementary Figs. 1–4). These interactions suggest that Thymoquinone may influence ligand binding, cleavage, or other crucial steps in Notch signalling. The variations in binding modes across the receptors offer insights into its potential selectivity. Taken together, these findings provide a detailed understanding of how certain compounds can interact with the Notch receptors and offer new avenues for research into the development of targeted therapies.

The Epigallocatechin gallate ligand appeared to have the best docking results via MDS based on the Vina score of all tested bio-compounds. Docking results of the compounds (Epigallocatechin gallate, Piperine, Gingerol, Thymoquinone) with the P53 and NOTCH (1–4) proteins showed a ball and stick representation of the ligands, with cartoon backbone representation of target proteins displayed in Fig. 5. The overall docked score for each pair is shown in Supplementary Table 1 and 2, which gave information on cavity size, cavity volume, centre (x,y,z) axis, amino acid residue numbers, and docked vina score that provided in-depth information about each protein–ligand pair and their corresponding figures as shown in Supplementary Figs. 1–5. As the Epigallocatechin gallate (Green tea) ligand showed the highest scoring function, we performed further CB-dock experiments with the target proteins to analyse the various protein–ligand complexes (Table 5).

**Table 5:** Results of CB-dock of green tea ligand with target proteins (P53, NOTCH 1–4)

S. No	Target	Ligand	Vina score	Curpocket ID	Cavity volume (A $^\circ$ 3)	Centre	Contact residues
1	P53	Green tea	− 9.2	C1	1146	− 8,16, − 26	GLU287 ASN288 LEU289 ARG290 LYS291 LYS292 GLY293 GLU294 PRO295 HIS296 HIS297 ASP324 GLY325 GLU326 TYR327 LEU348 GLU349 ASP352 ALA353 GLY356
2	NOTCH 1	Green tea	− 8.1	C1	4802	28, − 2,18	SER784 GLN789 THR790 ASN791 ILE792 ASN793 ALA796 ASP808 ASP809 ARG1841 HIS1847 ASP1873 ASN1875 VAL1876 ARG1877 THR1883 SER1919 ASN1922
3	NOTCH 2	Green tea	− 8.1	C2	1661	− 14, 34, − 24	HIS122 LEU124 GLU130 CYS131 THR132 PRO690 CYS691 ARG692 ALA695 CYS706 CYS708 PRO709 GLU710 GLY711 PRO712 HIS713 HIS714 PRO715 SER716 CYS717 TYR718
4	NOTCH 3	Green tea	− 9.4	C3	1502	44, − 7, − 8	ASN1438 ASN1455 PHE1456 ASP1457 GLU1464 ARG1465 THR1466 CYS1467 GLU1472 GLU1492 GLN1533 ARG1534 ALA1537 ILE1538 LEU1539 ARG1540 TYR1610 LEU1614 ARG1619 ASP1621
5	NOTCH 4	Green tea	− 7.5	C1	1084	− 23, − 39, 22	MET795 GLY796 PHE797 LYS805 LEU806 ARG807 PRO808 ASP812 SER813 PRO814 ARG816 GLN826 GLY827 PRO828 CYS830 GLY838 GLY839 SER840 GLN842

### Molecular dynamics simulation

UCSF Chimera was used to conduct a molecular dynamic simulation (MDS) study to understand the conformational changes that occur over time, along with the mode of interface with the receptor and its ligand. We chose one phytochemical receptor complex based on the highest scoring function and ran a 2500-step MDS on them for 100 ns. Next, using the TIP3PBOX solvate water model in a cubic box with box size 1, the system was set automatically for runs, and when all the systems were neutralized with the addition of Cl ions, energy minimization was done to eliminate any steric incompatibilities within the system. After that, a 1-nano second NVT and NPT simulation was run to keep the system’s pressure at 1 atm and temperature at 300 K. The complexes were then put forward for a 100-ns MDS, and the coordinates were saved once per femtosecond. The Root Mean Square Deviation (RMSD) plot for each protein–ligand complex was analysed following the simulation. Results of the MDS study and refinement process indicated that the complexes had been optimized for refinement, and the overall energy of the complex has also been stabilized, as structures presented good RMSD scores.

**Fig. 6:**
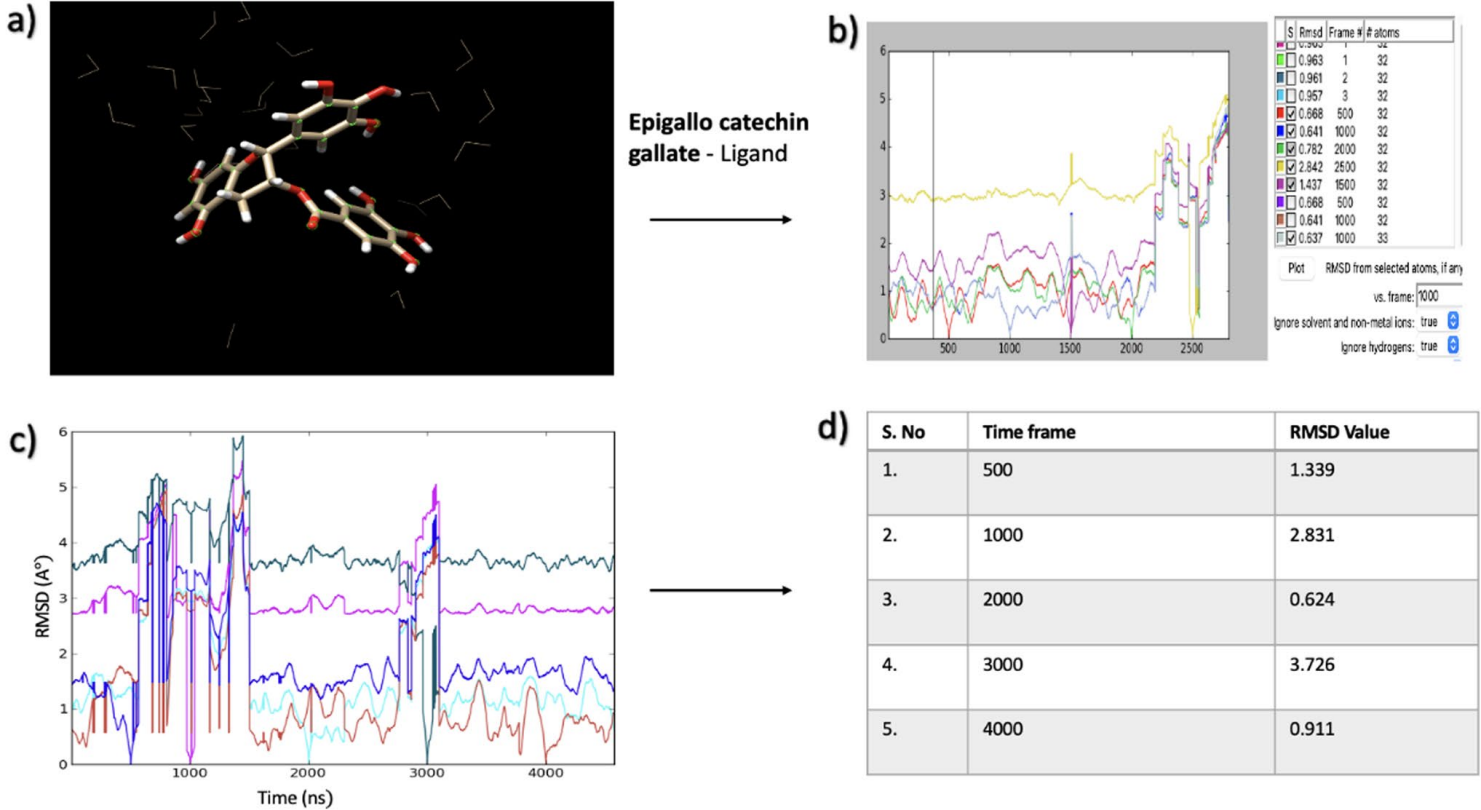
**a** Green tea active bio-compound Epigallocatechin gallate during MDS study. **b** RMSD plot filters based on time frame. **c** Root mean square deviation (RMSD) plot for ligand structure after three steps (Minimization, equilibration, production)

**Fig. 7:**
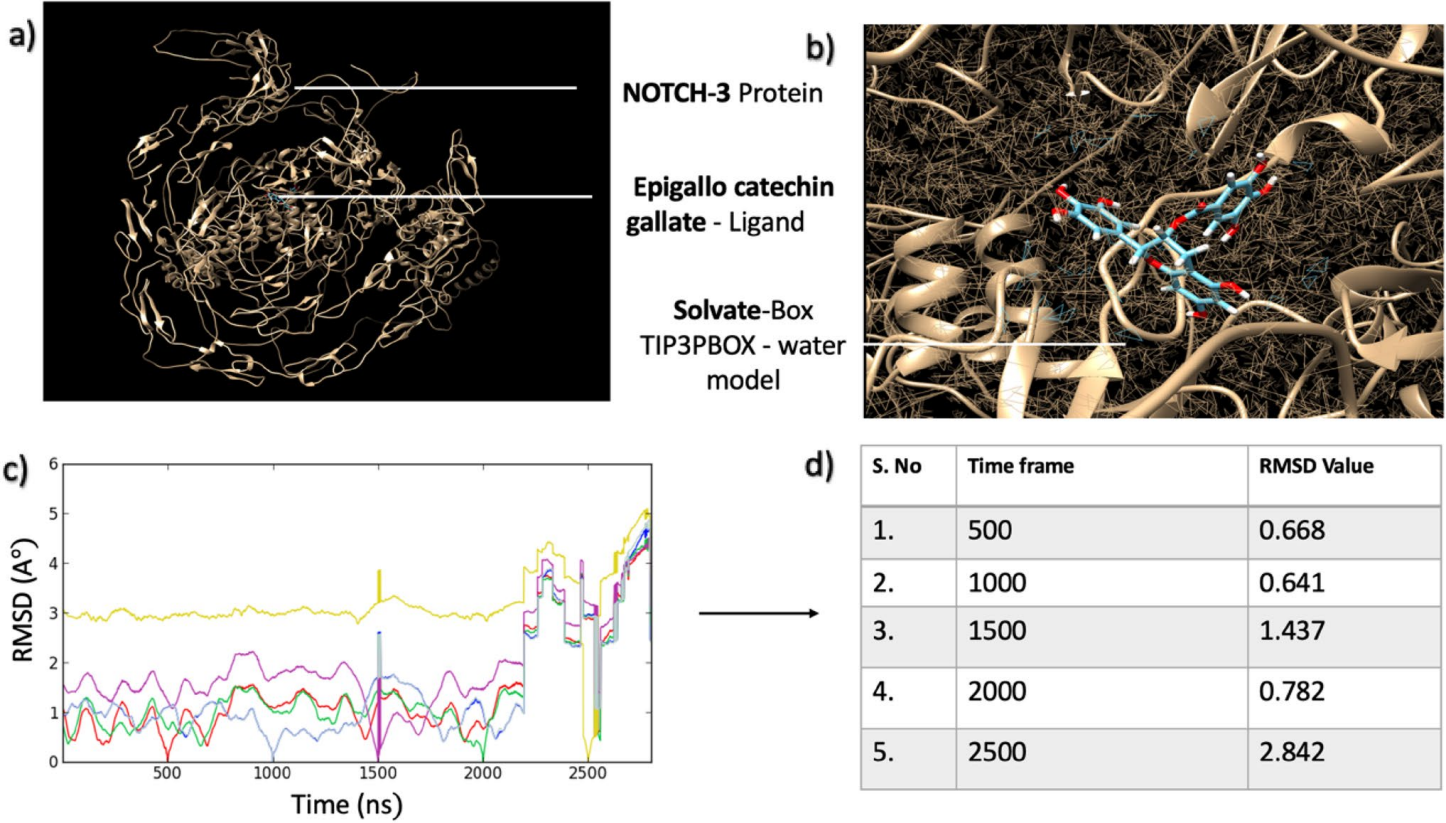
**a** NOTCH3 protein-Green tea active bio-compound Epigallocatechin gallate before MDS study, **b** Target-ligand complex during minimization process, **c** Root mean square deviation (RMSD) plot for target-ligand complex after four steps (Minimization, equilibration, production)

From our observations, Epigallocatechin gallate with Notch 3 protein appeared to be the most stable complex due to an observed higher overall energy than the other tested phytochemicals. The stability of the refined structure was encapsulated by the RMSD, which showed various oscillations present in the complex during the MDS and checked to see if the simulation had been balanced. The contacts and ligand-binding posture energy variability values of the Green tea ligand bound to Notch 3 was reported to have an RMSD score of 0.66, indicating minor changes following the refining of the complex. The molecular dynamic study was separately performed for Epigallocatechin gallate ligand alone as seen in Fig. 6 and with the NOTCH3 protein complex as shown in Fig. 7. The results showed that in different time frames, the RMSD value tended to change indicating the phytochemical’s stability in the complex. When the ligand was tested separately, the RMSD values of each time frame were mostly within the acceptable range (with the exception of a few outliers that exceeded the threshold value). However, the RMSD values were all within the acceptable range in the protein–ligand complex. This indicated that the complex was likely to be stable and could be used as a basis for further wet-lab experiments (Fig. 7).

## Discussion and future scope

Drug discovery and development are a lengthy, expensive, and risky process that can take up to 10–15 years to complete. On average, each new drug that’s approved for clinical use costs over $1–2 billion to develop and 90% of drug development ventures often lead to clinical failures [133]. According to clinical trial data from 2010 to 2017, the main reasons for these failures were lack of clinical efficacy, unmanageable toxicity, poor therapeutic properties, limited commercial needs, and poor strategic planning [91, 92]. Early assessment of a compound’s pharmaceutical properties is crucial for improving the effectiveness of the research and development process[134, 135]. However, the conventional methods currently used to assess ADMET characteristics are costly, time-consuming, and typically need substantial animal experimentation [136]. The preferred method for early drug discovery is now based on computer modelling approaches for predictive models [137]. Analysing a compound’s ADMET qualities in the early stages of drug development allows for the preliminary elimination of compounds with unfavourable ADME-Tox properties. In addition to using the predictive models as a guide for hit/lead compounds, the identification and structural optimisation of the compound can also yield extensive information that further elucidates its pharmaceutical and chemical properties. Existing in silico, in vitro, and in vivo ADME-Toxicity data should be supported by intelligent integration in the era of big data to better direct drug discovery pathways.

The interaction between protein and ligand is crucial in the design of drugs. Studies of protein–ligand interactions offer a theoretical framework for developing and identifying novel therapeutic targets [138, 139]. The interactions between Notch receptors and plant-derived ligands explored in this study have provided promising computational information on candidate compounds for use in breast cancer therapy. This includes developing a framework for comprehensive analyses of the mechanism of interactions, signalling, and binding affinity between phytochemicals and Notch Receptors via computational techniques. Our main work was to examine herbal phytochemicals that may theoretically and effectively interact with the NOTCH 1,2,3,4, and P53 proteins, increasing cancer cell death and reducing tumour spread against breast cancer cells. Only 4 of the 12 ADMET-screened compounds (Epigallocatechin gallate, Piperine, Gingerol, Thymoquinone) were found to be viable, and these were the ones that underwent molecular docking. Several of these phytochemicals were excluded due to a lack of .sdf/.mol structures available, or parsing errors occurred when they were subjected to molecular docking research. Thus, only four of the phytochemicals shortlisted were used for the molecular docking analysis.

The highest negative scoring function (higher negative vina score/binding energy) appeared to be the ideal preference as it showed better binding affinity between protein–ligand pairs [96, 97]. The binding energy discovered in the phytochemicals explored in this study was between − 9.4 and − 5.1 kcal/mol, which agreed with the ideal and standard binding energies. Our analysis revealed that ALA161, LEU194, CYS238, TYR236, ILE195, THR253 in P53 protein, along with ALA2006 and ARG 1824 in Notch 1 protein, LEU1807, ALA1806, LEU1805, SER1804, and THR1802 in Notch 2 protein, ASP404, GLN406, and GLN1601 in Notch 4 protein were promising residues for therapeutic targets. In addition, Epigallocatechin gallate, a ligand derived from green tea, showed potential as a compound for inhibiting the Notch 3 Receptor. Although the Notch 3 protein did not appear to interact with the Epigallocatechin gallate ligand, in silico docking analyses showed a high docking score/binding affinity, meaning further protein–ligand complex validation appears promising for potential therapeutic discovery [140, 141].

Creating therapeutic lead molecules using natural bioproducts through in silico modelling could save substantial time in creating new therapeutic products [142]. Clinical studies and wet-lab experimentation for validation are the logical next step in establishing these phytochemicals as new avenues of treatment for breast cancer and other cancers in general. Future drug development processes will take less time and resources due to the commercial computational tools that are readily available for calculating the pharmacological drug properties of candidate molecules. Findings from extensive in silico workflows will be important in selecting lead phytoconstituents for additional drug discovery steps. Within the current literature, the interest in herbal therapeutic compounds with the potential to provide resistance to breast cancer in humans is increasing. Herbal compounds with medicinal properties have been widely utilized for treatments of various diseases, such as diabetes, blood pressure, cancer, heart-related ailments, neurodegenerative diseases, and gastric issues [143]. There is growing evidence that herbally derived compounds provide a promising approach towards preventing and inhibiting breast cancer [144]. To advance our knowledge of using natural herbal compounds in breast cancer treatment and prevention, more research is required to explore the potential anti-breast cancer effects of these various compounds.

This study aimed to identify safe and efficient phytochemicals with the potential to work alongside signalling proteins to disrupt the cancer cycle. By screening several phytochemicals derived from Indian herbs, the in silico workflow developed in the study was able to uncover lead compounds for breast cancer therapy. Four phytochemicals, namely Epigallocatechin gallate, Piperine, Gingerol, and Thymoquinone, showed substantial binding with P53 and NOTCH proteins. These phytochemicals are also found in various other Indian herbs in combinational form, which presents the potential for further discovery. The current in silico investigation for putative P53 and NOTCH protein inhibitors from Indian herbs presented novel targets for the interaction with breast cancer biomarkers. This has the potential to direct further clinical research into the safe and efficient development of anti-cancer therapies.

### Supplementary Information

Below is the link to the electronic supplementary material.

Supplementary file1 (DOCX 1448 kb)

## Data Availability

The authors confirm that the data supporting this study’s findings is either available within the article or can be shared on reasonable request.
