# Supplementary material for: Analysing potent biomarkers along phytochemicals for breast cancer therapy: an in silico approach
Source: Breast Cancer Res Treat. 2023 Sep 20;203(1):29–47. doi: 10.1007/s10549-023-07107-7 (PMC10771382; doi:10.1007/s10549-023-07107-7)
Supplement: Supplementary file 1 — Supplementary file1 (DOCX 1448 kb) [file 10549_2023_7107_MOESM1_ESM.docx]

**SUPPLEMENTARY INFORMATION**

Supplementary figure 1: Docked poses of P53 protein with phytochemicals


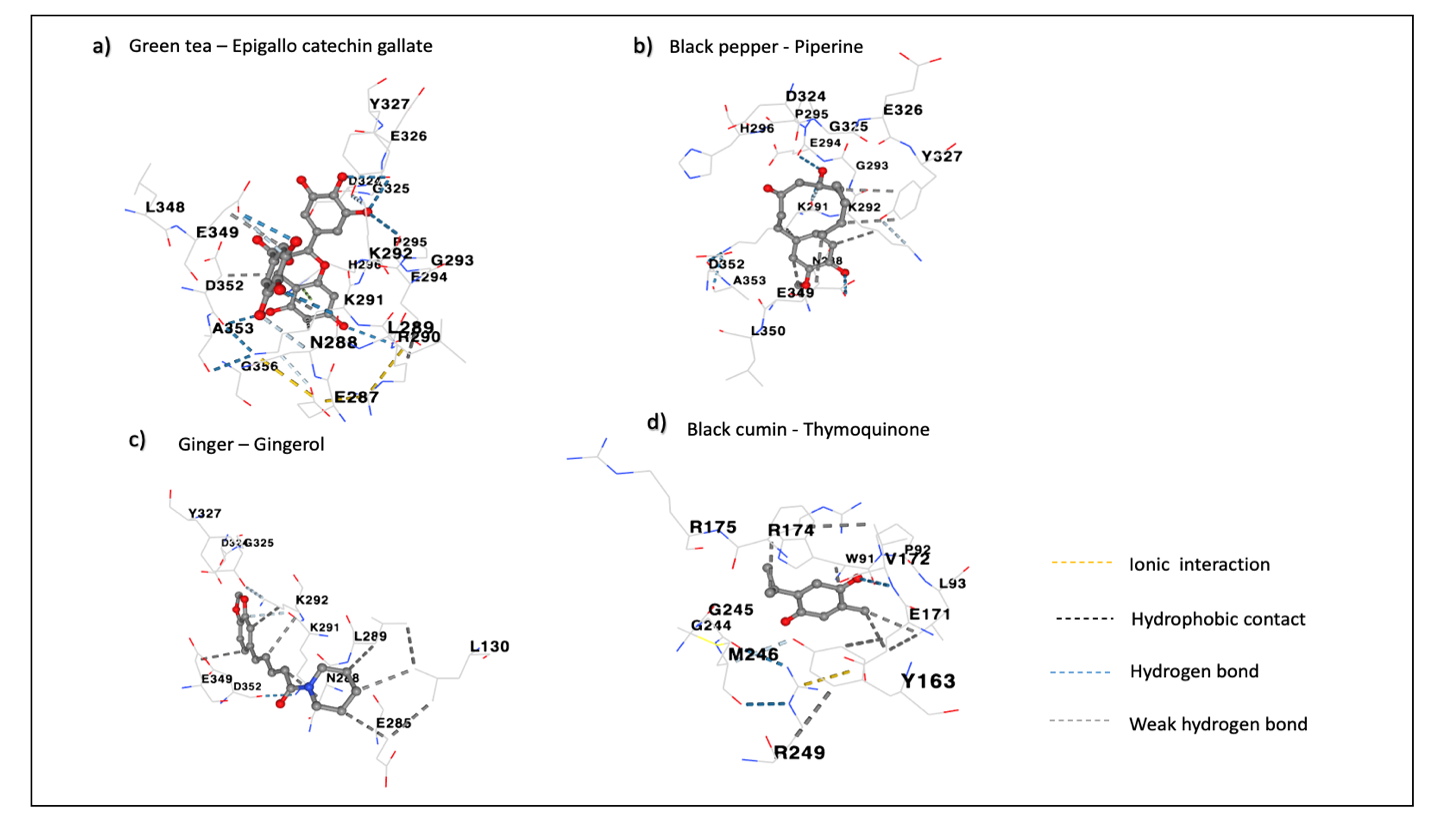


Supplementary figure 2: Docked poses of NOTCH 1 protein with phytochemicals


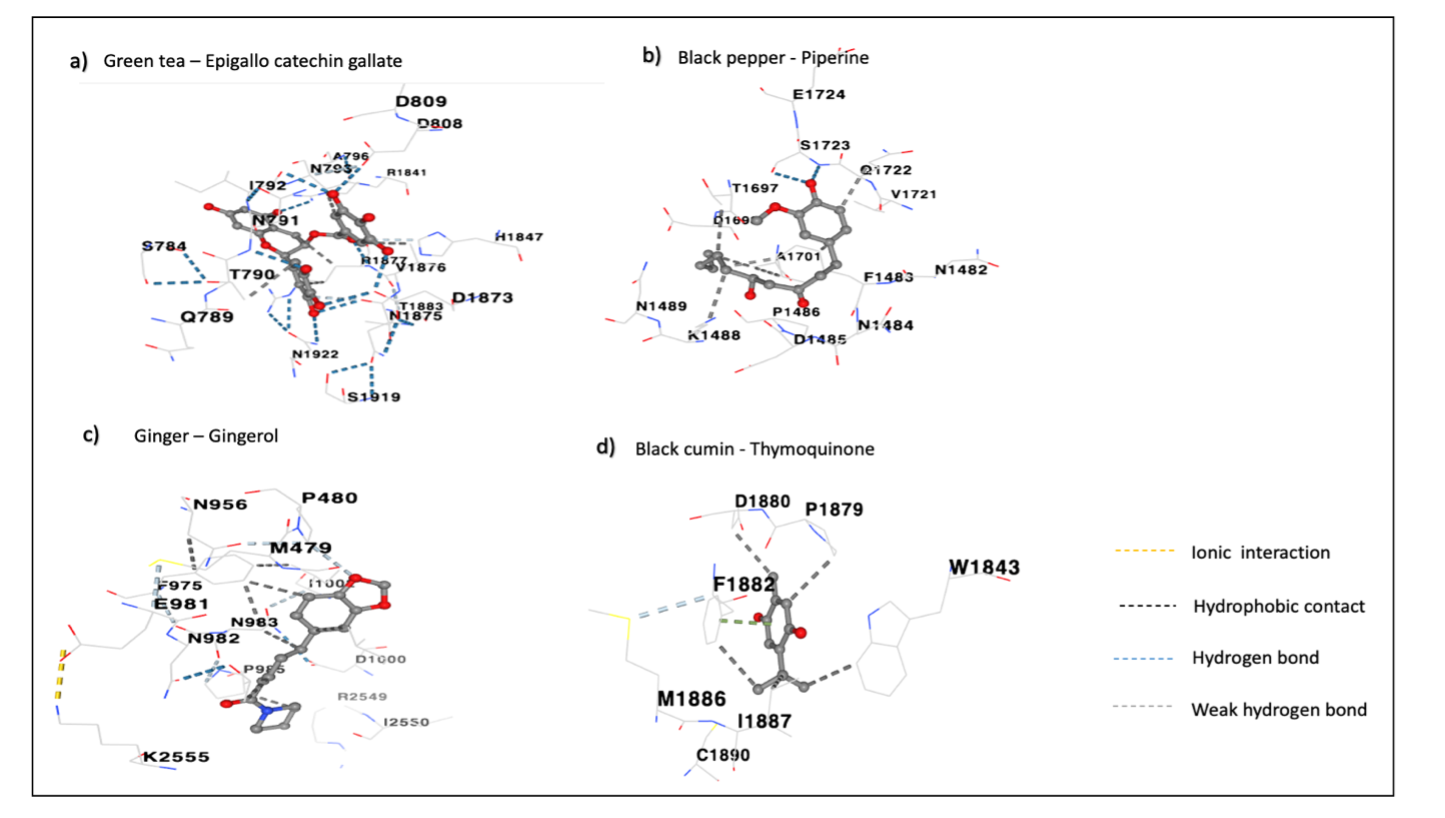


Supplementary figure 3: Docked poses of NOTCH 2 protein with phytochemicals


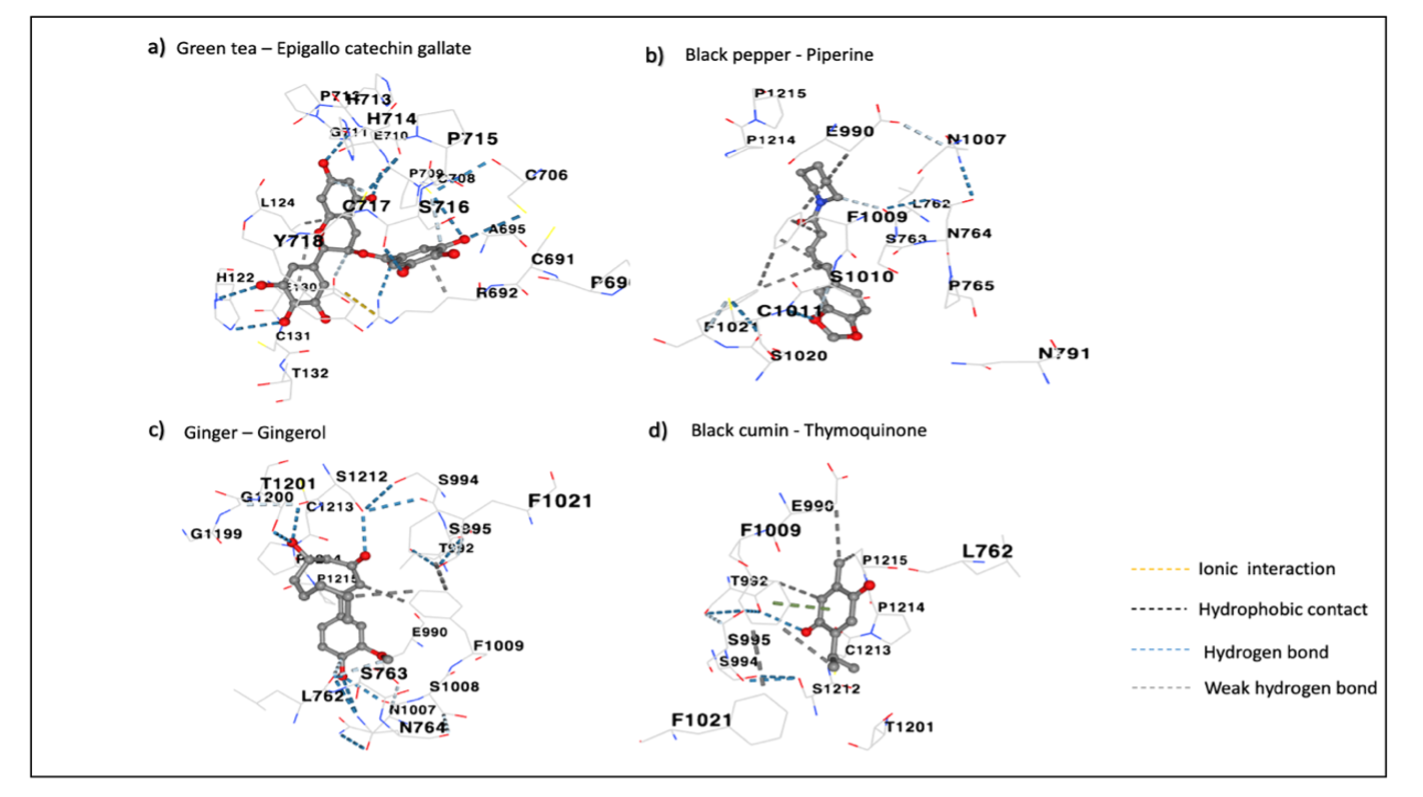


Supplementary figure 4: Docked poses of NOTCH 3 protein with phytochemicals


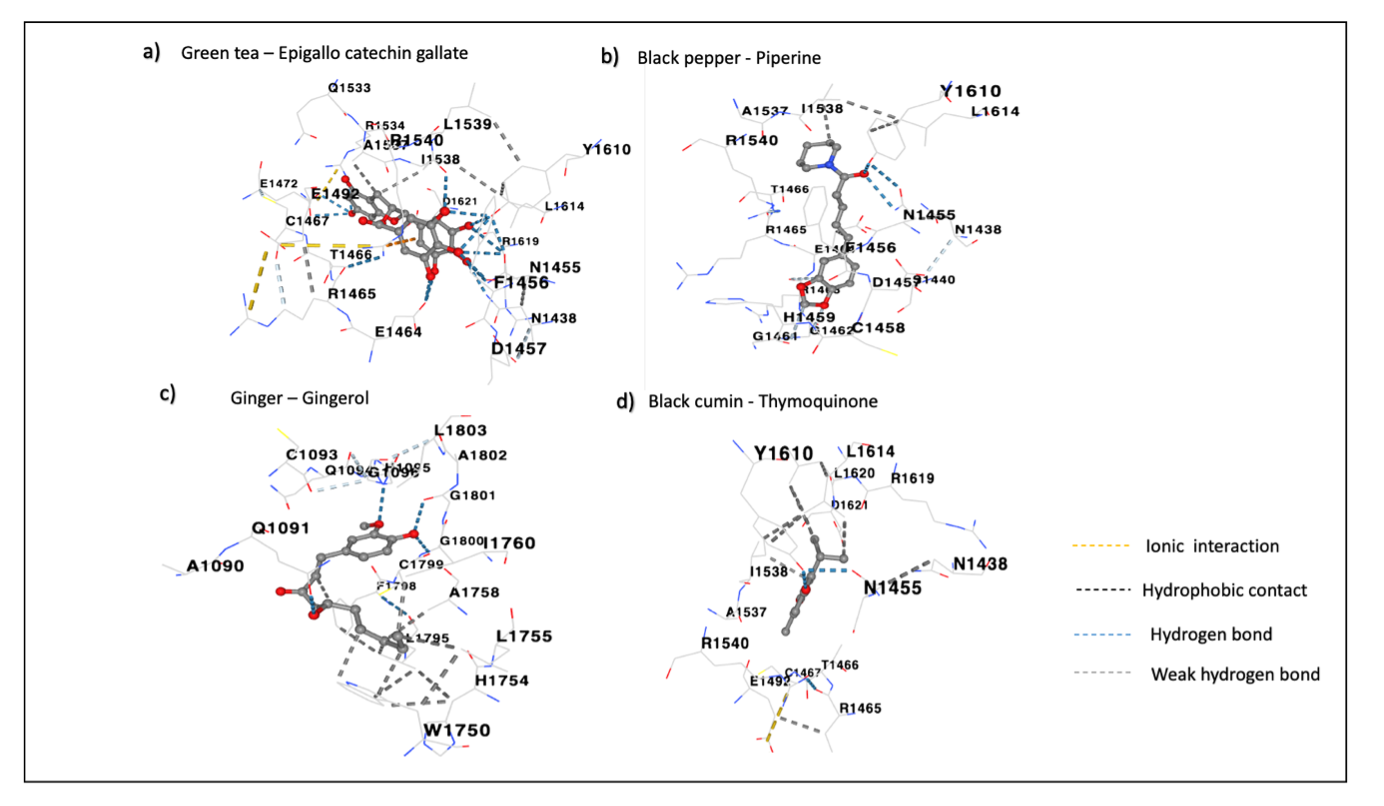


Supplementary figure 5: Docked poses of NOTCH 4 protein with phytochemicals


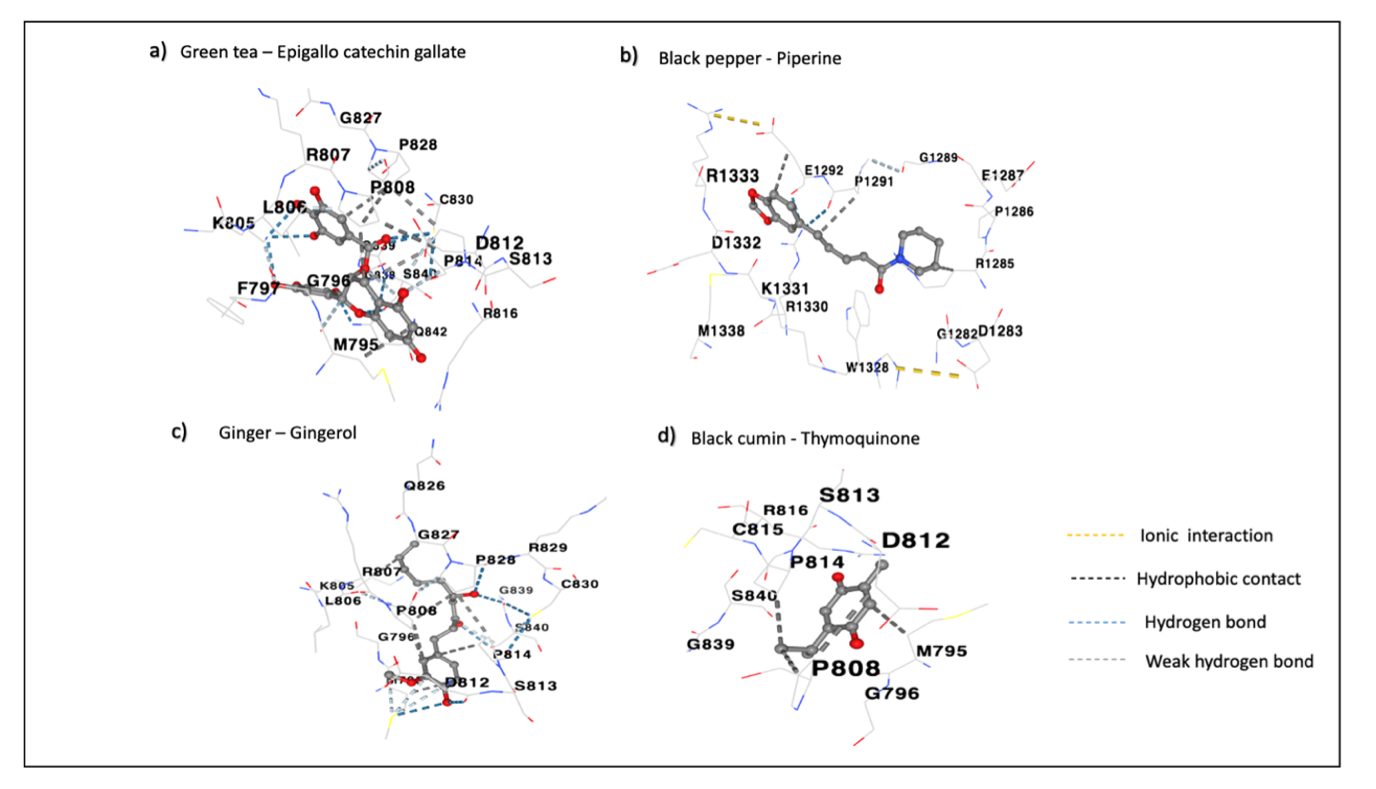


Supplementary Table 1: CB Docking results of P53 protein with different phytochemicals.

| **S.NO** | **TARGET -PROTEIN** | **LIGAND-PHYTOCHEMICALS** | **VINA SCORE** | **CURPOCKET ID** | **CAVITY VOLUME (A^3)** | **CENTER** | **CONTACT RESIDUES** |
| --- | --- | --- | --- | --- | --- | --- | --- |
| 1. | P53 | Green tea | -9.2 | C1 | 1146 | -8,16, -26 | GLU287 ASN288 LEU289 ARG290 LYS291 LYS292 GLY293 GLU294 PRO295 HIS296 HIS297 ASP324 GLY325 GLU326 TYR327 LEU348 GLU349 ASP352 ALA353 GLY356 |
| 2. | P53 | Black pepper | -7.2 | C1 | 1146 | -8, 16, -26 | LEU130 GLU285 ASN288 LEU289 LYS291 LYS292 ASP324 GLY325 TYR327 GLU349 ASP352 |
| 3. | P53 | Ginger | -6.0 | C2 | 340 | 8, -9, -5 | TRP91 PRO92 LEU93 TYR163 GLU171 VAL172 ARG174 ARG175 GLY244 GLY245 MET246 ARG249 |
| 4. | P53 | Black cumin | -5.6 | C1, C5 | 1146 | -8, 16, -26  3, 4, 11 | ASN288 LYS291 LYS292 GLY293 GLU294 PRO295 HIS296 ASP324 GLY325 GLU326 TYR327 LEU348 GLU349 LEU350 LYS351 ASP352 ALA353 |

Supplementary Table 2: CB Docking results of Notch (1-4) protein with different phytochemicals.

| **S.NO** | **TARGET -PROTEIN** | **LIGAND-PHYTOCHEMICALS** | **VINA SCORE** | **CURPOCKET ID** | **CAVITY VOLUME (A^3)** | **CENTER (X,Y,Z)** | **CONTACT RESIDUES** |
| --- | --- | --- | --- | --- | --- | --- | --- |
| 1. | NOTCH1 | Green tea | -8.1 | C1 | 4802 | 28, -2,18 | SER784 GLN789 THR790 ASN791 ILE792 ASN793 ALA796 ASP808 ASP809 ARG1841 HIS1847 ASP1873 ASN1875 VAL1876 ARG1877 THR1883 SER1919 ASN1922 |
| 2. | NOTCH1 | Black pepper | -7.2 | C2 | 1129 | -6, -28, 22 | THR466 ILE477 CYS478 MET479 PRO480 ASN956 PHE975 GLU981 ASN982 ASN983 PRO985 ASP1000 ILE1002 ARG2549 ILE2550 LYS2555 |
| 3. | NOTCH1 | Ginger | -5.7 | C3 | 932 | 47, 15, -24 | ASN1482 PHE1483 ASN1484 ASP1485 PRO1486 LYS1488 ASN1489 THR1697 ASP1698 ALA1701 VAL1721 GLN1722 SER1723 GLU1724 |
| 4. | NOTCH1 | Black cumin | -5.8 | C1 | 4802 | 28, -2, 18 | TRP1843 PRO1879 ASP1880 PHE1882 MET1886 ILE1887 CYS1890 |
| 5. | NOTCH2 | Green tea | -8.1 | C2 | 1661 | -14, 34, -24 | HIS122 LEU124 GLU130 CYS131 THR132 PRO690 CYS691 ARG692 ALA695 CYS706 CYS708 PRO709 GLU710 GLY711 PRO712 HIS713 HIS714 PRO715 SER716 CYS717 TYR718 |
| 6. | NOTCH2 | Black pepper | -7.3 | C1 | 1661 | 2, -2, 13 | LEU762 SER763 ASN764 PRO765 ASN791 GLU990 ASN1007 PHE1009 SER1010 CYS1011 SER1020 PHE1021 PRO1214 PRO1215 |
| 7. | NOTCH2 | Ginger | -5.4 | C1 | 1661 | 2, -2, 13 | LEU762 GLU990 THR992 SER994 SER995 PHE1009 PHE1021 THR1201 SER1212 CYS1213 PRO1214 PRO1215 |
| 8. | NOTCH2 | Black cumin | -5.6 | C1 | 1661 | 2, -2, 13 | LEU762 SER763 ASN764 GLU990 THR992 SER994 SER995 ASN1007 SER1008 PHE1009 PHE1021 GLY1199 GLY1200 THR1201 SER1212 CYS1213 PRO1214 PRO1215 |
| 9. | NOTCH3 | Green tea | -9.4 | C3 | 1502 | 44, -7, -8 | ASN1438 ASN1455 PHE1456 ASP1457 GLU1464 ARG1465 THR1466 CYS1467 GLU1472 GLU1492 GLN1533 ARG1534 ALA1537 ILE1538 LEU1539 ARG1540 TYR1610 LEU1614 ARG1619 ASP1621 |
| 10. | NOTCH3 | Black pepper | -8.7 | C3 | 1502 | 44, -7, -8 | ASN1438 SER1440 ASN1455 PHE1456 ASP1457 CYS1458 HIS1459 GLY1461 GLY1462 ARG1463 GLU1464 ARG1465 THR1466 ALA1537 ILE1538 ARG1540 TYR1610 LEU1614 |
| 11. | NOTCH3 | Ginger | -6.2 | C2 | 2714 | 8, -17, -33 | ALA1090 GLN1091 CYS1093 GLN1094 HIS1095 GLY1096 TRP1750 HIS1754 LEU1755 ALA1758 ILE1760 LEU1795 PHE1798 CYS1799 GLY1800 GLY1801 ALA1802 LEU1803 |
| 12. | NOTCH3 | Black cumin | -5.5 | C3 | 1502 | 44, -7, -8 | ASN1438 ASN1455 ARG1465 THR1466 CYS1467 GLU1492 ALA1537 ILE1538 ARG1540 TYR1610 LEU1614 ARG1619 LEU1620 ASP1621 |
| 13. | NOTCH4 | Green tea | -7.5 | C1 | 1084 | -23, -39, 22 | MET795 GLY796 PHE797 LYS805 LEU806 ARG807 PRO808 ASP812 SER813 PRO814 ARG816 GLN826 GLY827 PRO828 CYS830 GLY838 GLY839 SER840 GLN842 |
| 14. | NOTCH4 | Black pepper | -7.0 | C5 | 683 | 49,24,7 | HIS1264 GLY1282 ASP1283 ARG1285 PRO1286 GLU1287 GLY1289 PRO1291 GLU1292 TRP1328 ARG1330 LYS1331 ASP1332 ARG1333 MET1338 |
| 15. | NOTCH4 | Ginger | -5.9 | C1 | 1084 | -23, -39, 22 | MET795 GLY796 LYS805 LEU806 ARG807 PRO808 ASP812 SER813 PRO814 GLN826 GLY827 PRO828 ARG829 CYS830 GLY839 SER840 |
| 16. | NOTCH4 | Black cumin | -5.1 | C1 | 1084 | -23, -39, 22 | MET795 GLY796 PRO808 ASP812 SER813 PRO814 CYS815 ARG816 GLY839 SER840 |
